# Patient selection and outcome in low-grade glioma surgery

**DOI:** 10.3389/fonc.2025.1703756

**Published:** 2025-11-27

**Authors:** Margret Jensdottir, Ole Solheim, Alba Corell, Eddie de Dios, Tora Dunås, Alexander Fletcher-Sandersjöö, Sasha Gulati, Klas Holmgren, Francesco Latini, Ruby Mahesparan, Peter Milos, Alice Neimantaite, Henrietta Nittby Redebrandt, Lars Kjelsberg Pedersen, Rickard L. Sjöberg, Björn Sjögren, Gregor Tomasevic, Øystein Vesterli Tveiten, Tomás Gómez Vecchio, Maria Zetterling, Jiri Bartek, Asgeir S Jakola

**Affiliations:** 1 Department of Clinical Neuroscience, Karolinska Institutet, Stockholm, Sweden; 2 Department of Neurosurgery, Karolinska University Hospital, Stockholm, Sweden; 3 Department of Neuromedicine and Movement Science, Faculty of Medicine and Health Sciences, Norwegian University of Science and Technology, NTNU, Trondheim, Norway; 4 Department of Neurosurgery, St. Olavs Hospital, Trondheim University Hospital, Trondheim, Norway; 5 Institute of Neuroscience and Physiology, Sahlgrenska Academy, University of Gothenburg, Gothenburg, Sweden; 6 Department of Neurosurgery, Sahlgrenska University Hospital, Gothenburg, Sweden; 7 Department of Neurosurgery, University Hospital of Northern Sweden, Umeå, Sweden; 8 Department of Clinical Sciences, Neuroscience, Umeå University, Umeå, Sweden; 9 Department of Medical Sciences, Section of Neurosurgery, Uppsala University Hospital, Uppsala, Sweden; 10 Department of Clinical Medicine, Faculty of Medicine, University of Bergen, Bergen, Norway; 11 Department of Neurosurgery, Haukeland University Hospital, Bergen, Norway; 12 Department of Neurosurgery, Linköping University Hospital, Linköping, Sweden; 13 Department of Biomedical and clinical Sciences, Linköping University, Linköping, Sweden; 14 Department of Clinical Sciences, Skåne University Hospital, Lund, Sweden; 15 Department of Neurosurgery, Skåne University Hospital, Lund, Sweden; 16 Department of Neurosurgery, University Hospital of North Norway, Tromsø, Tromsø, Sweden; 17 Department of Neurosurgery, Copenhagen University Hospital Rigshospitalet, Copenhagen, Copenhagen, Denmark

**Keywords:** extent of resection, glioma, low-grade glioma, neurological deficits, neurosurgery, oncology, surgical outcomes

## Abstract

**Background and objectives:**

Maximal safe resection is the cornerstone of diffuse low-grade glioma (dLGG) management, although epidemiological data are limited. We aim to explore surgical selection, techniques, and outcomes in a population-based cohort.

**Materials and methods:**

This study utilized a multi-center case series (9 out of 10 neurosurgical departments in Norway and Sweden) of all adults (≥18 years) with histopathologically verified supratentorial dLGG, WHO grade 2, undergoing primary surgery from 2012-2017. Complications within 30 days and neurological outcomes at 3 months were assessed. Pre- and postoperative MRIs were reviewed centrally, blinded to patient outcome and center.

**Results:**

Of 517 included patients, 217 (41.7%) were female, and the mean (SD) age was 44.5 (15.0) years. Biopsy only was performed in 119 (23.0%) patients (13.8-38.9% across centers), and 398 (77.0%) underwent resection (61.1-86.2%). Intraoperative neurophysiological monitoring (IONM) was used in 142 (35.7%, 0-58.7%) resections. The biopsy-only patients were older (52.7 years vs. 42.1 years, P<.001), had larger tumors (56.6 ml vs. 31.9 ml, P<.001), and these tumors were more often eloquently located (86.6% vs. 56.5%, P<.001). The median (IQR) extent of resection (EOR) was 82.9% (63.3-97.7%), 69.7-100.0% across centers. The median (IQR) residual tumor was 4.6 ml (0.5-19.9 ml), 0.0-14.1 ml across centers. Age and histopathology were the most important predictors of EOR. New/worsened neurological deficits occurred in 165 patients (41.5%), 23.1-66.7% across centers, and persisted in 19 (4.8%, 0-22.7%) at 3 months after surgery. A complication was observed in 87 patients (21.4%, 0-31.7%), with 12 patients (3.1%, 0-9.8%) having grade III-IV complications.

**Conclusions:**

We found that surgical selection was associated with age, tumor size, and location. The median EOR in a population-based cohort was 83%, with age and tumor biology being significant predictors. EOR did not correlate with higher risks or worse neurological outcomes. We provide an epidemiological perspective demonstrating a variation in surgical selection and techniques reflecting persistent controversy in dLGG management.

## Introduction

Maximal safe resection remains a key therapeutic approach for diffuse low-grade gliomas (dLGG) and provides significant survival benefits (1–5). Upfront surgery is recommended for diagnostic and therapeutic purposes. Biopsy only is an adverse prognostic factor for overall survival (6–8). Investigating surgery selection at a population level can help us understand the context of the surgical literature.

The reported risk of post-operative neurological worsening after resection varies, ranging from 2-40% (9–13). Intraoperative neurophysiological monitoring (IONM) reportedly increases tumor resection while minimizing risks of permanent neurological deficits (14, 15).

Although IONM is widely accepted in specialized centers, epidemiological data are limited. Generalizing findings from highly specialized hospitals to broader clinical settings is difficult (16, 17). Hence, controversies persist regarding the influence of surgical selection and techniques on outcome and presumed resectability of dLGG.

The aim of this multi-center study was to investigate the correlation between surgical selection and use of surgical adjuncts and the effectiveness and safety of surgery on a population level. Specifically, we focused on short term outcomes: extent of resection (EOR), adverse events, and postoperative neurological deficits. Secondarily, we aimed to explore inter-hospital variations.

## Materials and methods

### Ethics and approvals

The regional committee of Western Sweden (EPN reference 705/17) and The Regional Committee for Medical and Health Research Ethics in Central Norway (REC reference 2017/1780) approved the study with a waiver of informed consent.

### Patient population and definition of cohort

This study is part of a Scandinavian multicenter project, including all neurosurgical departments performing glioma surgery in Norway and Sweden. The publicly funded health care systems, with compliant regional referral, practically eliminate any referral bias. The study design, population, data collection, and variables have been described previously (18).

All adults (≥18 years) with histopathologically confirmed supratentorial dLGG, World Health Organization (WHO) grade 2, undergoing primary surgery (biopsy or resection) between January 1, 2012, and December 31, 2017, were included in the database. Tumors with anaplastic features (WHO grade 3-4) were excluded. Histopathological classification was based on the classification system in use at time of surgery (WHO 2007 or WHO 2016).

This study includes all patients from centers providing radiological data for central review (9/10).

### Study variables

Patient characteristics, diagnostic work-up, and surgical techniques were retrospectively retrieved from medical records and pseudonymized data entered in electronic case report forms (CRFs). IONM parameters were extracted from surgical records and neurophysiological reports and data were collected on mapping techniques, whether the patients was awake or under general anesthesia, indication for mapping (language, motor, sensory, visual, or other), and whether passive monitoring with MEP was performed. Preoperative Magnetic Resonance Imaging (MRI) was analyzed locally for tumor location, laterality, contrast enhancement, and presumed eloquent area, defined according to Chang et al. (3).

MRIs were extracted from the respective hospitals PACS (picture archiving and communication system, Sectra™), with volumetrics performed centrally. Trained raters segmented the tumor volumes on pre- and postoperative MRI from T2-weighted or FLAIR images or, in exceptional cases, contrast-enhanced T1-weighted images, using 3D Slicer software (19). All segmentations were validated by a neurosurgeon (ASJ) with significant experience in dLGG management and research, including volumetric assessment. Raters were blinded to patient clinical status and treating center at the time of segmentation.

EOR was calculated as [(preoperative tumor volume − postoperative tumor volume)/preoperative tumor volume] × 100%. Gross total resection (GTR) was defined as 100% EOR/0.0 ml of tumor remnant.

New postoperative neurological deficits were recorded in detail from the retrospective review of medical records. Functional outcome was assessed at 3 months with persistent deficits considered permanent. Permanent deficits were classified as minor (i.e., limited impact on daily life: can work, drive, travel, perform physical activities, and live a rather normal socioprofessional life) or major (i.e., impact on daily life: limitation with respect to work, drive, travel, and/or physical activities and thus can´t live a normal socioprofessional life). Adverse events occurring within 30 days postoperatively were categorized using the Landriel-Ibanez Classification (LIC) (20). The LIC grades severity of complications are as follows: grade I being any non-life-threatening event not expected postoperatively (e.g. new onset seizure, pneumonia, or urinary tract infection); grade II is any complication requiring intervention (e.g. lumbar drainage or re-operation due to cerebrospinal fluid leakage); grade III is any life-threatening complication (e.g. brain swelling, acute postoperative hematoma, or hydrocephalus); and grade IV is fatal. We considered grade I complications mild, grade II moderate, and grade III severe. This is a reasonable scaling since neurological deficits were reported separately, avoiding underreporting major deficits in the grade I category.

### Statistics

Statistical analysis was performed in IBM SPSS Statistics for Windows, Version 25.0 (Armonk, NY: IBM Corp) and R (version 4.1.2). Normality of continuous data was explored using the Shapiro-Wilk test. Central tendencies are presented as means ± SD or median and interquartile range (IQR) for normally distributed and skewed data, respectively. Comparison between groups was performed using Mann-Whitney U test for non-parametric data and independent two samples t-test for parametric data. Chi-square tests or Fishers exact test were used in intergroup comparisons of categorical variables, and categorical data are expressed as numbers and percentages. All tests were two-sided and statistical significance was set at *P* <.05. Scatterplots were used to assess relationships in the data. A univariable and forced-entry multivariable logistic regression was used to identify predictors of postoperative permanent neurological deficits. To determine predictors of EOR (ranging from 0 to 100%), univariable and forced-entry zero-one inflated beta regression (ZOIB) analyses were employed. ZOIB was preferred based on the clustering nature of EOR around 100%.

## Results

### Patient characteristics and clinical presentation

**Figure 1:**
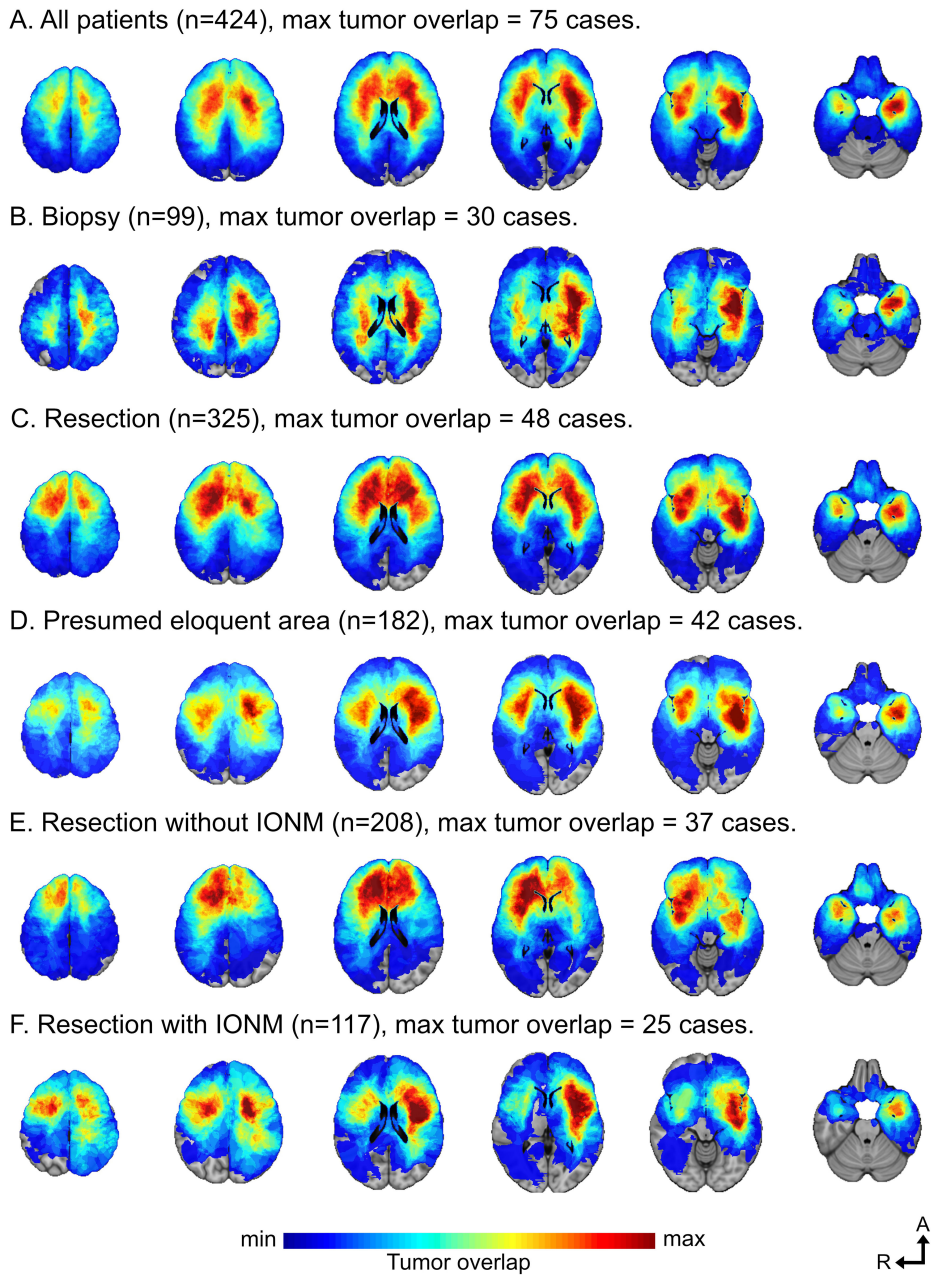
**(A-F)** A location heatmap showing spatial distribution for all tumors **(A)**. Separate heatmaps for the tumor locations in patients selected for biopsy **(B)**, resection **(C)**, presumed eloquent area **(D)**, resection without IONM **(E)**, and resection with IONM **(F)**. Figures based on available tumor segmentations on Montreal Neurological Institute (MNI) space (axial slices 50, 35, 20, 5, -10 and -25 mm). The scale of tumor overlap was adjusted to the maximum overlap for each group **(A-F)**.

We included 517 patients with a summary of preoperative patient characteristics, tumor classification, intraoperative techniques, and outcomes in **Tables 1**–**3** and **Supplementary Table 1**. The average age was 44.5 years and 41.7% were female. Seizures were the most common presenting symptom (N = 305, 59.0%). A majority had a Karnofsky Performance Score (KPS) of 90-100 (N = 390, 74.9%). For an overview of tumor location, see **Table 1** and **Figures 1A-F**. Tumors were presumed to involve an eloquent area in 328 patients (63.4%), ranging from 33.3-68.2% across centers.

**Table 1:** Summary of epidemiological patient characteristics.

Characteristics	Total (n=517)	Biopsy (n=119)	Resection (n=398)	*P* value
Age in years, mean (SD)	44.5 (15.0)	52.7 (14.7)	42.1 (14.2)	<.001
Female, No. (%)	217 (41.7)	53 (44.5)	164 (41.2)	.52
Preoperative symptoms, no. (%)				
Seizure	305 (59)	70 (58.8)	235 (59)	.97
Cognitive deficit	57 (10.9)	25 (21)	32 (8)	<.001
Motor deficit	66 (12.7)	30 (25.2)	36 (9)	<.001
Language deficit	42 (8.1)	21 (17.6)	21 (5.3)	<.001
Visual deficit	38 (7.3)	14 (11.8)	24 (6.0)	.04
Headache/ICP-related symptoms	107 (20.5)	19 (16)	88 (22)	.02
Asymptomatic/incidental	65 (12.5)	7 (5.9)	57 (14.3)	.01
Preoperative KPS score, no. (%)				
90-100	388 (75.0)	75 (63.0)	313 (78.6)	.01
80	72 (13.9)	22 (18.5)	50 (12.6)	
≤70	57 (11.0)	22 (18.5)	35 (8.8)	
Diagnostic radiological work-up, no. (%)				
MRI	517 (100)	119 (100)	398 (100)	
Spectroscopy	131 (25.3)	31 (26.1)	100 (25.1)	
PET	35 (6.8)	9 (7.6)	26 (6.5)	
Tumor volume (ml), median (IQR)	35.9 (63.5)	56.6 (70.5)	31.9 (57.5)	<.001
Missing data, No.	23	6	17	
Contrast enhancement on MRI	157 (30.4)	46 (38.7)	111 (27.9)	.03
Patchy/Diffuse/Weak	102 (19.7)	31 (26.1)	71 (17.8)	
Nodular	41 (7.9)	12 (10.1)	29 (7.3)	
Ring-like	14 (2.7)	3 (2.5)	11 (2.8)	
Main tumor location, no. (%)				
Frontal	283 (54.7)	44 (37)	239 (60.1)	<.001
Temporal	107 (20.7)	26 (21.8)	81 (20.4)	
Parietal	51 (9.9)	16 (13.4)	35 (8.8)	
Occipital	13 (2.5)	6 (5.0)	7 (1.8)	
Insula	39 (7.5)	9 (7.6)	30 (7.5)	
Central/deep/basal ganglia/thalamus	24 (4.6)	18 (15.1)	6 (1.5)	
Laterality, no. (%)				
Left	265 (51.4)	61 (51.3)	204 (51.3)	<.001
Right	230 (44.4)	44 (37)	186 (46.7)	
Bilateral/midline	22 (4.2)	14 (11	8 (2)	
Multifocal, No. (%)	38 (7.4)	27 (22.7)	11 (2.8)	<.001
Presumed eloquent location^a^	328 (63)	103 (86.6)	225 (56.5)	<.001
Preoperative functional imaging, no. (%)				
Any Advanced imaging	267 (51.6)	30 (25.2)	237 (59.5)	<.001
Functional MRI, fMRI	141 (27.1)	10 (8.4)	131 (32.9)	
Diffusion Tensoir Imaging, DTI	219 (42)	25 (21)	194 (48.7)	
Transcranial Magnetic Stimulation, nTMS	97 (18.6)	2 (1.7)	95 (23.9)	
Histopathology, no. (%)				
Astrocytoma	270 (52.2)	85 (71.4)	185 (46.5)	<.001
Oligodendroglioma	183 (35.4)	20 (16.8)	163 (41)	<.001
Oligoastrocytoma^b^	64 (12.4)	14 (11.8)	50 (12.6)	.82
IDH status, no. (%)				
Mutated	245 (47.4)	31 (26.1)	214 (53.8)	<.001
Wild type	75 (14.5)	37 (31.1)	38 (9.5)	<.001
Not assessed	197 (38.1)	51 (42.9)	146 (36.7)	.22

ICP, intracranial pressure; IDH, isocitrate dehydrogenase; KPS, Karnofsky Performance Status; MRI, magnetic resonance imaging; PET, positron emission tomography, IQR; Interquartile range.

a According to Chang et al. Preoperative prognostic classification system for hemispheric low-grade gliomas in adults. J Neurosurg. Nov 2008;109 (5):817-24.

b Of those 62 pt diagnosed according to WHO 2007 criteria and 2 pt according to WHO 2016 criteria.

**Table 2:** Surgical, oncological, and functional outcomes for patients undergoing resection.

	Total (n=398)	No IONM (n=256)	IONM (n=142)	*P* value
Tumor remnant (ml), median (IQR)	4.6 (0.5-19.9)^a^	3.4 (0.3-18.7)^b^	6.2 (0.8-22.9)^c^	.09
0.0 ml, GTR No. (%)	79 (19.8)	56 (21.9)	23 (16.2)	.14
0.1-4.9 ml, No. (%)	108 (27.1)	71 (27.7)	37 (26.1)	
5-14.9 ml, No. (%)	68 (17.1)	38 (14.8)	30 (21.1)	
≥ 15 ml, No. (%)	109 (27.4)	67 (26.2)	42 (29.6)	
Volumetric EOR in %, median (IQR)	82.9 (63.3-97.7)^a^	85.3 (63.4-98.8)^b^	80.4 (62.7-94.7)^c^	.19
100% (GTR), No. (%)	79 (19.8)	56 (21.9)	23 (16.2)	.15
90-99.9%, No. (%)	57 (14.3)	40 (15.6)	17 (12)	
80-89.9%, No. (%)	59 (14.8)	32 (12.5)	27 (19)	
70-79.9%, No. (%)	49 (12.3)	33 (12.9)	16 (11.3)	
<70%, No. (%)	120 (30.2)	72 (28.1)	48 (33.8)	
New/worse neurological deficits, No. (%)^d^	165 (41.5)	65 (25.7)	100 (70.4)	<.001
Language	76 (19.1)	36 (14.2)	40 (28.2)	.001
Motor	87 (21.9)	32 (12.6)	55 (38.7)	<.001
SMA syndrome	19 (4.8)	3 (1.2)	16 (11.3)	<.001
Visual	20 (5)	12 (4.7)	7 (4.9)	.89
Cognitive	20 (5)	13 (5.1)	7 (4.9)	.98
Sensory	12 (3)	3 (1.2)	9 (6.3)	.01
Parietal lobe syndrome	2 (0.5)	0	2 (1.4)	.05
Permanent major neurological deficit, No. (%)^d,e^	19 (4.8)	7 (2.7)	12 (8.5)	.97
Language	5 (1.3)	3 (1.2)	2 (1.4)	.56
Motor	11 (2.8)	4 (1.6)	7 (4.9)	.92
Visual	2 (0.5)	0	2 (1.4)	.15
Cognitive	4 (1.0)	3 (1.2)	1 (0.7)	.81
Parietal lobe syndrome	3 (0.8)	0	3 (2.1)	.12

EOR, extent of resection; GTR, gross total resection; IONM, intraoperative neurophysiological monitoring and mapping; IQR, interquartile range; SMA, supplementary motor area.

a Missing values=34, therefore numbers may not sum to group totals and percentages do not add to 100%.

b Missing values=24, therefore numbers may not sum to group totals and percentages do not add to 100%.

c Missing values=10, therefore numbers may not sum to group totals and percentages do not add to 100%.

d Patient wise. All neurological deficits are reported, resulting in a higher total number than reported under the compilation since each patient can have more than one deficit.

e Permanent major deficit, Neurological deficit persistent at 3 months and having impact on daily life.

**Table 3:** Preoperative work up, surgical techniques, and perioperative treatment for tumors in a presumed eloquent area vs not eloquent area.

Resection	Total	Eloquent	Not eloquent	*P* value
	(n=398)	(n=225)	(n=173)	
Age, mean (SD)	42.1 (14.2)	42.8 (14.3)	41.0 (14.0)	.21
Female, No. (%)	164 (41.2)	87 (38.7)	77 (44.5)	.25
Preoperative KPS, no. (%)				
90-100	313 (78.6)	168 (74.7)	143 (83.6)	.02
80	50 (12.6)	36 (16.0)	14 (8.2)	
70 or less	35 (8.8)	21 (9.3)	14 (8.2)	
Tumor volume (ml), median (IQR)	31.9 (12.4-69.9)	39.6 (21.2-79.7)	19.9 (7.1-60.1)	<.001
Any focal deficit, No. (%)	89 (22.4)	67 (29.8)	21 (12.1)	<.001
Advanced imaging^a^, No. (%)	237 (59.5)	167 (74.2)	69 (40.4)	<.001
Neuronavigation, No. (%)	353 (88.7)	198 (88.0)	155 (89.6)	.74
Microsurgical techniques, No. (%)	368 (92.5)	209 (92.9)	159 (91.9)	.93
Ultrasonic aspirator, No. (%)	244 (61.3)	148 (65.8)	96 (55.5)	.05
IONM, No. (%)	142 (35.7)	122 (54.2)	20 (11.6)	<.001
Intraoperative imaging, no. (%)				
Ultrasound	224 (56.6)	127 (56.4)	96 (55.4)	.96

KPS, Karnofsky Performance Status; IONM, intraoperative neurophysiological monitoring and mapping; IQR, interquartile range.

a Advanced imaging: functional magnetic resonance imaging (fMRI), diffusion tensor imaging (DTI), navigated transcranial magnetic stimulation (nTMS).

### Preoperative work-up and surgical selection

Biopsy only was performed in 119 patients (23%, 13.8%-38.9% across centers) and 398 (77%, 61.1-86.2%) underwent resection. Advanced functional imaging (functional Magnetic Resonance Imaging (fMRI), Diffusion Tensor Imaging (DTI), or navigated Transcranial Magnetic Stimulation (nTMS)) was used in 267 patients (51.6%, 25.0-94.0%), (**Table 1**). IONM was employed in 142 (35.7%, 0-58.7) resected patients and only in four (3.4%) of the biopsied patients.

The patients undergoing biopsy had a cluster of negative prognostic factors including older age, lower KPS scores, larger and multifocal tumors, higher proportion of contrast enhancing tumors, and more frequent involvement of presumed eloquent brain regions. (**Table 1**) Further, the tumor location heatmaps demonstrate a more prominent subcortical infiltration in the biopsy cohort compared to more cortical overlap in resected patients (**Figures 1B, C**). In patients with available isocitrate dehydrogenase (IDH) mutation status, IDH wild-type (wt) tumors were more common. The IDH status also showed a correlation to contrast enhancement with a significantly higher proportion of contrast enhancement in IDH wt status tumors compared to IDH mutated, 32.0% and 22.4% respectively (p<0.001). Complications related to biopsies are listed in **Supplementary Table 2**.

### Surgical techniques and outcome

Neuronavigation and microsurgical techniques were used in most resections (N = 353, 88.7% and N = 368, 92.5%, respectively). Intraoperative imaging was solely performed with ultrasound (N = 224, 56.6%, 11.5%-100% across centers). Intraoperative mapping or monitoring was performed in 142 resections (35.7%, 0-58.7% across centers). Awake surgery was performed in 62 patients (15.6%, 0-28.6% across centers), asleep mapping only in 80 patients (20.1%, 0-34.6% across centers), and three patients (0.8%) were exclusively monitored during asleep resection without any mapping with direct electrical stimulation (DES) (**Supplementary Table 1**).

Given the significance of IONM in dLGG surgery and the challenges of tumors located in presumed eloquent areas, we analyzed cases in depth with respect to these factors. The differences in baseline between cases in a presumed eloquent vs non-eloquent area are presented in **Table 3**, and **Supplementary Table 1** provides a detailed description of indications and type of IONM (asleep/awake/indication for mapping/monitoring). In short, significantly more patients with a tumor in a presumed eloquent area were investigated with advanced imaging techniques and selected for resection with IONM compared to patients with tumors in a presumed non-eloquent area (**Table 3**).

**Figure 2:**
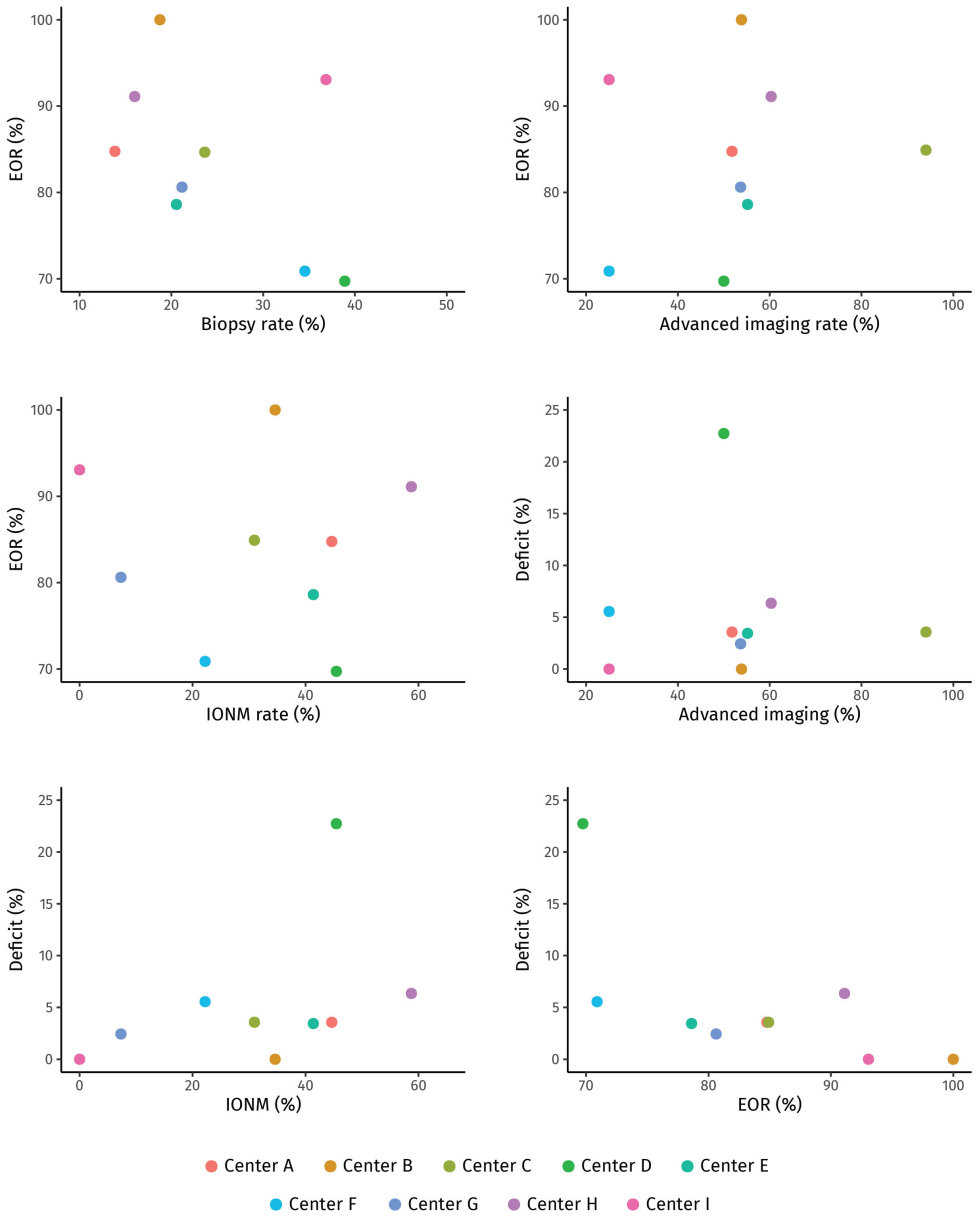
**(A-F)** Scatterplots displaying relationship between key outcome parameters for all resected tumors by patient centers (dots), each center represented with a different color. **(A)** The relationship between proportion of biopsy and median EOR. **(B)** The relationship between proportion of advanced imaging and median EOR. **(C)** The relationship between proportion of IONM and median EOR. **(D)** The relationship between proportion of advanced imaging and proportion of permanent major neurological deficit. **(E)** The relationship between proportion of IONM and proportion of permanent major neurological deficit. **(F)** The relationship between median EOR and proportion of permanent major neurological deficits.

**Table 2** outlines the overall surgical outcome. The median tumor remnant was 4.6 ml (0.0-14.1 across centers) and the median EOR was 82.9% (69.7-100.0% across centers [demonstrated in **Supplementary Figure 1**)], with 79 patients (19.8%, 4.5-50.0%) achieving GTR. Comparing centers, we found no correlation between the EOR and either biopsy rates, the frequency of preoperative advanced imaging, or the use of IONM (**Figures 2A-C**). A similar pattern was observed in the subgroup of eloquent area tumors (**Supplementary Figures 2D, E**). In a subgroup analysis of all tumors in a presumed eloquent area, a significantly higher EOR was observed with IONM (77.0% versus 67.6%, p=0.019) and a smaller median tumor remnant (8.1 ml versus 12.5 ml, p=0.062).

**Figure 3:**
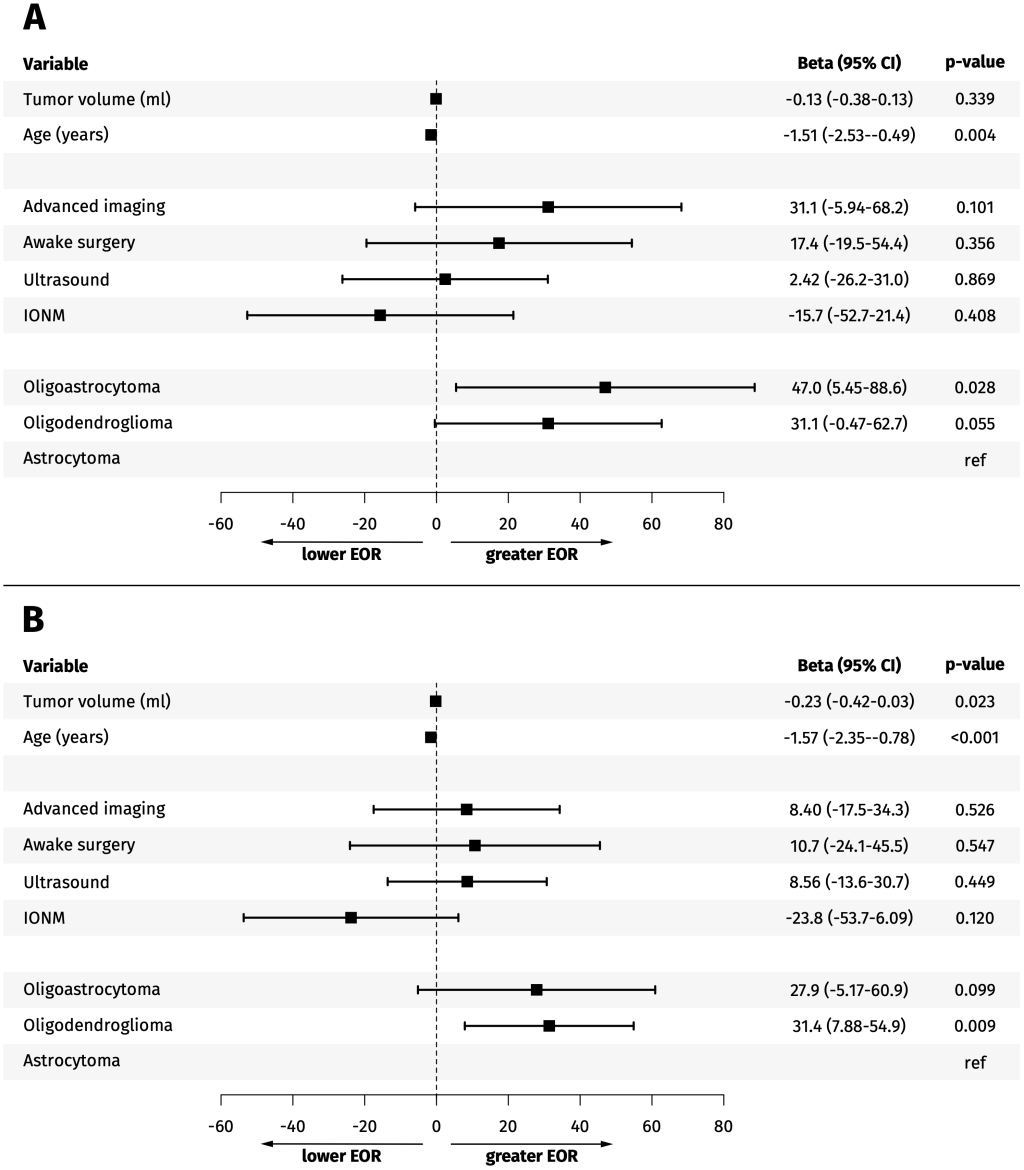
**(A, B)** Forest plot. Multivariable analysis of predictors of extent of resection (EOR) for resected tumors in a presumed eloquent area **(A)** and all resected tumors **(B)**.

We found that age predicted a lower EOR with a -1.57 or -1.51 unit decrease in EOR for each increased year of age for all tumors and tumors in eloquent areas, respectively (**Figure 3**). Histopathology significantly affected EOR, with oligodendroglioma predicting a higher EOR (**Figure 3**). The median EOR (IQR) for oligodendroglioma was 86.2% (69.1-98.3%) and was 79.4% (56.7-98.5%) for astrocytoma. A summary of a univariable and multivariable analysis of possible predictors of EOR is provided in **Supplementary Table 3**.

### Functional outcome

New or worsened neurological deficits occurred in 165 patients (41.5%, 23.1-66.7% across centers), with only 19 patients (4.8%, 0.0-22.7%) suffering permanent major deficits and no correlation between deficits and EOR (**Figure 2F**).

Comparing resections with or without IONM showed a higher frequency of permanent major deficits (8.5% vs 2.7%) and postoperative seizures (9.2% vs 3.2%) in patients resected with IONM. A comprehensive table of neurological deficits and outcomes related to indications for IONM is available in **Supplementary Tables 4, 5**.

Comparing centers, we found no correlation between neurological deficits and different imaging practices or use of IONM (**Figures 2D, E**, **Supplementary Figures 2D, E**). Nor could we identify any predictors of permanent major deficits (**Supplementary Table 3**).

To further analyze any association between outcome and the use of IONM, we plotted the percentage of EOR versus percentage of permanent major deficits for all tumors and tumors in eloquent areas and grouped by resection with or without IONM for each center (**Supplementary Figure 3**). We observed a lower percentage of permanent major deficits with an increased EOR with the use of IONM in tumors in eloquent areas. Contrarily, a higher rate of permanent major deficits was observed with a lower EOR, despite use of IONM.

Surgical complications occurred in 87 resected patients (21.9%, 0.0-31.7% across centers). A detailed description is found in **Supplementary Table 6**. A moderate (Grade II) or severe (Grade III) complication occurred in 32 patients (8.0%, 3.6-12.2%). Severe complications were mostly postoperative hemorrhage (e.g., cavity hematoma and epidural hematoma), occurring in 11 patients (2.8%,1.6-9.8%). A single patient (0.3%) suffered from a Grade IV complication, with urosepsis as the suspected cause of death.

## Discussion

This population-based cohort demonstrates that patients selected for biopsy only have a distinct profile compared to those undergoing resection, characterized by worse tumor burden, older age, and lower functional status. Further, we found that median EOR is approximately 83% without clear predictors other than age and histology. We also describe the epidemiological perspective on short-term outcomes, with transient neurological deficits and minor permanent deficits dominating.

In this study, which was not restricted to IDH-mutated tumors, IDH wildtype (IDH wt) tumors were identified as a confounder, given their association with location, contrast enhancement, age, treatment, and prognosis according to the WHO classification applicable during the study period (21, 22). As expected, IDH wt tumors were more often biopsied. However, surgical decision making is done without knowledge of dLGG subclassification, mostly influenced by age, cognitive function, KPS, tumor location, and imaging findings as negative prognostic factors (23, 24). More prominent subcortical infiltration, shown in the location heat maps, correlated with higher rates of cognitive decline and neurological symptoms. This then suggests its potential as a marker for surgical planning, as proposed by Ng et al (25),, identifying clinical, radiological, and oncological markers associated with effective onco-functional balance. This population-based setting demonstrates the biopsy group as heavily selected. Comparing biopsy with resection from a single institution, in registries etc., is intrinsically flawed unless capturing unique situations, as in Jakola et al (2, 26), who compared regions favoring different surgical strategies.

We investigated relationships between preoperative assessments, surgical adjuncts, and short-term outcomes to identify factors that could aid surgical decision-making. Particular attention was given to IONM due to its significant role in dLGG treatment within the surgical community.

As expected, a correlation was found between using IONM and advanced imaging for presumed eloquent tumors. However, a substantial variation between centers in the frequency of use is in concordance with previous studies, which show great heterogeneity in the imaging practice for dLGG in Europe, reflecting limited evidence of effectiveness (27, 28). Observed variations did not affect EOR or rates of neurological complications. This contrasts with a previous randomized trial by Wu et al (29),, who found an improved outcome with presurgical planning with DTI. Functional imaging can identify eloquent areas prior to surgery and reportedly the preoperative use of nTMS correlated with improved outcome (30, 31). This discrepancy with our findings could be attributed to selective use of advanced imaging modalities in cases where higher risks are anticipated, potentially leading to confounding by indication.

The predilection for use of IONM in presumed eloquent areas and convincing evidence at the time in favor of the use of IONM to maximize safe resection (14, 15, 32, 33) corresponds to a yearly increase in use of IONM during the study period (18). This increasing trend and learning curve for the use of IONM probably explains the overlapping location in the heatmap for biopsied patients and those resected with IONM. Only 15.6% underwent awake surgery. Given landmark studies on the contribution of awake mapping for improved functional outcome and survival (14, 34, 35) it could be argued that underutilization has occurred. We noted a variance across centers in the use of awake surgery (0-28.6%), and the yearly increase in the use of IONM may reflect that a transition was ongoing throughout these years. The reason for the low percentage of awake surgeries, particularly at some centers, may be due to institutional culture or surgeon preferences, a knowledge gap, or a more protracted learning curve outside highly specialized centers (13, 36–38). However, from our sample, it must still be emphasized that we do not see a clear signal of improved outcomes in centers more frequently performing surgeries under the guidance of IONM in general or awake surgery specifically.

The importance of awake surgery to achieve long-term functional outcomes has recently been demonstrated in a landmark paper by Ng et al. (39) In a large cohort of 600 patients with a newly diagnosed IDH mutant grade 2 glioma, awake resection contributed to a median survival of 20 years and a median preservation of KPS ≥80% of 14.7 years after surgery.

The EOR and tumor remnant achieved in this population compares favorably to previous reports. Capelle et al (40), in a French multicenter database, the largest cohort of dLGG to date, reports a median EOR of 76.8%, median tumor remnant of 10 ml, and 11.9% undergoing GTR. Wijnenga et al (41), report a similar median EOR of 76.1%, 10.95 ml median tumor remnant, and 15.4% GTR in a cohort of 228 patients from two Dutch centers. On the other hand, Hervey-Jumper et al (16), in a cohort of 392 patients from a large volume specialized center reports a higher median EOR (92.0%), lower median tumor remnant (2.6 ml), and 30.9% GTR, which may reflect a combination of being an experienced high-volume center and patient selection. The factors identified to correlate with EOR were tumor location and histopathology. The importance of histopathology has previously been reported and eloquent regions are more frequently found in proximity to IDH-mutated astrocytomas (42). The importance of tumor location for EOR underscores the need for objective preoperative evaluation to identify eloquent area location and estimating resectability (43, 44). This is essential for surgical decision-making where improved selection aims to avoid high-risk surgery in patients with a low chance of achieving a meaningful EOR.

The importance of surgical selection and maximal safe resection has been further consolidated by a recent publication by The Response Assessment in Neuro-Oncology (RANO) resect group (45). This is an international and multidisciplinary group, with the aim to standardize the measurement of extent of resection and its impact on survival in adult diffuse glioma, classified by WHO 2021 classification. They stress the importance of quantification of extent of resection, such as the objective volumetric assessment performed in our study, to facilitate comparison between studies.

Based on a comprehensive review of the relevant literature, Karschnia et al. have established an algorithm to help estimate expected survival gains from different degrees of resection, considering tumor subtype, patient factors, risk of deficits, etc., to guide which patients are likely to benefit most from more aggressive surgery. For slower-growing tumor types, such as IDH-mutant astrocytomas and oligodendrogliomas, these benefits unfold over years/decades (45).

We observed a significantly higher incidence of new deficits in patients who underwent resection with IONM, in concordance with the literature reporting a common occurrence of neurological worsening in presumed eloquent tumors (15, 46). Fortunately, there was no significant difference between the subgroups in terms of permanent major deficits. Additionally, no correlation between increased extent of resection (EOR) and a higher frequency of deficits or adverse events was observed. Nor did we find significant variation across centers.

The challenge is to maximize the resection in highly eloquent areas. In our subgroup analysis we found a significantly higher EOR in presumed eloquent areas when using IONM without a higher risk of permanent deficits. This finding contrasts with other studies suggesting that more extensive resection correlates with worse outcomes in eloquent areas. Coburger et al (47),, in a German multicenter study evaluating the outcome of surgery for dLGG in eloquent areas, reported 10% permanent severe deficits and increased deficits with a higher EOR. However, other studies have emphasized the tumor location in eloquent areas as a possible risk factor for survival due to less extensive resection, further emphasizing the controversies regarding the resectability of dLGG (34, 48, 49).

We observed a lower proportion of permanent major deficits with more complete resection and, contrarily, more deficits with lower extent of resection despite the use of IONM, although mainly driven by an outlier. This reflects the importance of patient selection since some patients will not benefit from resection regardless of the use of IONM due to tumor location and degree of white matter infiltration. These patients should be identified prior to surgery to avoid unnecessary risks associated with less meaningful attempts of surgical resection (43). Improvement in detecting subcortical infiltration in preoperative imaging could aid in surgical selection.

Most reported permanent deficits were considered minor, although 4.8% had deficits with an impact on their daily life. The comparison of our results to the literature is somewhat hampered due to the lack of use of standardized reporting systems.

We demonstrate that achieving a high EOR is feasible in population-based cohorts, without a high risk of major permanent deficits. These findings can aid in preoperative patient counseling by providing realistic information on expectations and risk profiles and facilitate shared decision-making.

## Limitations

An inherent limitation is the retrospective nature of the study. Additionally, a variance across centers of the frequency of presumed eloquent tumors, not expected in a population-based setting, reflects some risk of bias. However, the use of tumor maps displaying the true tumor location complements this to some extent.

Comparison of our results to the current literature, with molecularly defined subgroups according to the WHO 2021 classification, is somewhat hampered by the limited availability of IDH mutation status and the histopathological classification based on the system in use at time of surgery. However, IDH mutation status was available in 63.3% of the resected patients and only a minority were IDHwt. Therefore, excluding this limited number of patients would not significantly alter the results, and the risk of bias is considered low.

The major strength is the large population-based cohort with a multi-center approach across two countries with low rates of missing data. Further, a central review of radiological data, blinded to patient functional outcome and treating center, grants the study high generalizability.

## Conclusion

Age, tumor volume, and eloquence determine surgical selection. Age and histopathology, not surgical adjuncts, are the main predictors of EOR. Variation in surgical practices across centers in seemingly uniform health care systems reflect persistent controversies in dLGG management. Our results underscore the importance of careful patient selection to optimize dLGG treatment and standardized reporting from population-based settings to facilitate guidelines on surgical decision-making.

## Data Availability

The datasets presented in this article are not readily available because the data supporting findings of this study are available within the paper and its **Supplementary Materials**. The raw data of the study are not available due to restrictions in the ethical permits. Requests to access the datasets should be directed to margret.jensdottir@ki.se.

## Supplementary material

Supplementary Figure 1 Boxplot displaying EOR in each patient center.

Supplementary Figure 2 Scatterplots displaying relationship between key outcome parameters in resected tumors in presumed eloquent area by patient centers (dots), with each center represented with a different color. **(A)** The relationship between proportion of biopsy and median EOR. **(B)**The relationship between proportion of advanced imaging and median EOR. **(C)** The relationship between proportion of IONM and median EOR. **(D)** The relationship between proportion of advanced imaging and proportion of permanent major neurological deficit. **(E)** The relationship between proportion of IONM and proportion of permanent major neurological deficit. **(F)** The relationship between median EOR and proportion of permanent major neurological deficits.

Supplementary Figure 3 Bubble charts were used to illustrate the relationship between surgical and functional outcomes based on case volume and the use of IONM per center. The percentage of EOR versus percentage of permanent major deficits was plotted for all tumors and tumors in a presumed eloquent area and grouped by resection with or without IONM. Each dot represents a patient center, and the dot size represents case volume at the respective center (the scale for dot size is shown in figure legend).
