# Supplementary material for: Patient selection and outcome in low-grade glioma surgery
Source: Front Oncol. 2025 Nov 27;15:1703756. doi: 10.3389/fonc.2025.1703756 (PMC12696705; doi:10.3389/fonc.2025.1703756)
Supplement: Supplementary Figure 1 — Boxplot displaying EOR in each patient center. [file DataSheet1.docx]

**Supplementary material**

Variations in patient selection, surgical techniques, and surgical results in adult diffuse low-grade gliomas: a Scandinavian multi-center study

Margret Jensdottir, Ole Solheim, Alba Corell, Eddie de Dios, Tora Dunås, Alexander Fletcher-Sandersjöö, Sasha Gulati, Klas Holmgren, Francesco Latini, Ruby Mahesparan, Peter Milos, Alice Neimantaite, Henrietta Nittby Redebrandt, Lars Kjelsberg Pedersen, Rickard L. Sjöberg, Björn Sjögren, Gregor Tomasevic, Øystein Vesterli Tveiten, Tomás Gómez Vecchio, Maria Zetterling, Jiri Bartek Jr and Asgeir S. Jakola

**Supplemental Table 1.** Surgical selection – IONM, type and indication

|  | **Resection (n=398)** | **Indication for mapping^a^ (n=142)** | | | | | |
| --- | --- | --- | --- | --- | --- | --- | --- |
|  |  | Language | Motor | Sensory | Other parietal | Visual |  |
| Any IONM, No. (%) | 142 (35.7) | 43 (30.3) | 115 (81.0) | 20 (14.1) | 2 (1.4) | 6 (4.2) |  |
| Awake surgery^b^, No. (%) | 62 (15.6) | 43 (30.3) | 43 (30.3) | 6 (4.2) | 2 (1.4) | 3 (2.1) |  |
| Asleep mapping exclusively, No. (%) | 80 (20.1) | NA | 72 (50.7) | 14 (9.9) | 0 | 3 (2.1) |  |
| Continuous MEP´s, No (%) | 58 (14.6) | NA | 56 (39.4) | NA |  |  | |
| Continuous MEP´s exclusively, No (%) | 3 (0.8) | NA | 3 (2.1) | NA |  |  | |
| Resection stopped due to; No. (%) |  |  |  |  |  |  |  |
| Functional limits | 64 (45.1) | 25 (55.6) | 61 (53.0) | 7 (35.0) | 2 (100) | 2 (33.3) |  |
| Perceived GTR | 36 (25.4) | 10 (22.2) | 30 (26.1) | 8 (40.0) | 0 | 3 (50.0) |  |
| Not reported | 42 (29.5) | 10 (22.2) | 24 (20.9) | 5 (25.0) | 0 | 1 (16.7) |  |

Abbreviations: IONM, intraoperative neurophysiological monitoring and mapping; MEP, motor evoked potential; GTR, gross total resection; NA: not applicable.

^a^Several indications possible in each patient.

^b^Both awake and asleep mapping in 24 patients.

**Supplemental Table 2.** Adverse events within 30 days, according to Landriel Ibanez classification, in patients undergoing biopsy.

|  | **Biopsy (n=119)** |
| --- | --- |
| Any complication, No. (%) | 12 (10.1) |
| **Grade I** |  |
| New onset seizure / status epilepticus | 3 (2.5) |
| Worsened seizures | 1 (0.8) |
| Urinary tract infection | 2 (1.7) |
| Deep vein thrombosis | 1 (0.8) |
| Superficial wound infection | 1 (0.8) |
| Deep wound infection | 1 (0.8) |
| Postoperative cavity hematoma | 2 (0.8) |
| **Grade II** |  |
| Deep wound infection | 1 (0.8) |
| **Grade III** | 0 |
| **Grade IV** | 0 |
| Most severe complication, No. (%) |  |
| Grade I | 11 (9.2) |
| Grade II | 1 (0.8) |
| Grade III | 0 |
| Grade IV | 0 |

**Supplemental Table 3.A.-D.** Univariable and multivariable regression analysis.

**A. Zero-and-one inflated beta regression predicting EOR (0 – 100%) in all surgically treated tumors.**

| **Variable** | **Univariable model** | | **Forced entry multivariable model** | |
| --- | --- | --- | --- | --- |
|  | **Beta (95% CI)** | ***P* Value** | **Beta (95% CI)** | ***P* Value** |
| Age (years) | -1.37 (-2.14 – -0.60) | **< .001** | -1.57 (-2.35 – -0.78) | **< .001** |
| Awake surgery | 2.04 (-26.8 – 30.9) | .89 | 10.7 (-24.1 – 45.5) | .55 |
| IONM | -3.91 (-26.5 – 18.6) | .74 | -23.8 (-53.7 – 6.09) | .12 |
| Ultrasound | 9.06 (-13.1 – 31.3) | .42 | 8.56 (-13.6 – 30.7) | .45 |
| Advanced imaging^a^ | 10.0 (-12.6 – 32.6) | .39 | 8.40 (-17.5 – 34.3) | .53 |
| Tumor volume (cm^3^) | -0.24 (-0.44 - -0.04) | **.02** | -0.23 (-0.42 - -0.03) | **.02** |
| Histopathology |  |  |  |  |
| Oligoastrocytoma | 21.6 (-11.7 – 54.8) | .20 | 27.9 (-5.17 – 60.9) | .10 |
| Oligodendroglioma | 28.0 (4.37 – 51.5) | **.02** | 31.4 (7.88 – 54.9) | **.01** |
| Astrocytoma | reference | - | reference | - |

Abbreviations: IONM, intraoperative neurophysiological monitoring and mapping

^a^Advanced imaging: functional magnetic resonance imaging (fMRI), diffusion tensor imaging (DTI), navigated transcranial magnetic stimulation (nTMS)

**B. Zero-and-one inflated beta regression predicting EOR (0 – 100%) in all surgically treated eloquent tumors.**

| **Variable** | **Univariable model** | | **Forced entry multivariable model** | |
| --- | --- | --- | --- | --- |
|  | **Beta (95% CI)** | ***P* Value** | **Beta (95% CI)** | ***P* Value** |
| Age (years) | -1.28 (-2.23 – -0.33) | **.01** | -1.51 (-2.53 – -0.49) | **.01** |
| Awake surgery | 24.1 (-7.22 – 55.5) | .13 | 17.4 (-19.5 – 54.4) | .36 |
| IONM | 20.5 (-7.01 – 48.0) | .15 | -15.7 (-52.7 – 21.4) | .41 |
| Ultrasound | 7.57 (-20.5 – 35.6) | .60 | 2.42 (-26.2 – 31.0) | .87 |
| Advanced imaging^a^ | 42.0 (11.0 – 73.1) | **.01** | 31.1 (-5.94 – 68.2) | .10 |
| Tumor volume (cm^3^) | -0.20 (-0.45 – 0.04) | .11 | -0.13 (-0.38 – 0.13) | .34 |
| Histopathology |  |  |  |  |
| Oligoastrocytoma | 31.7 (-9.22 – 72.5) | .13 | 47.0 (5.45 – 88.6) | **.03** |
| Oligodendroglioma | 23.2 (-6.65 – 53.1) | .13 | 31.1 (-0.47 – 62.7) | .06 |
| Astrocytoma | reference | - | reference | - |

Abbreviations: IONM, intraoperative neurophysiological monitoring and mapping

^a^Advanced imaging: functional magnetic resonance imaging (fMRI), diffusion tensor imaging (DTI), navigated transcranial magnetic stimulation (nTMS)

**C. Univariable and forced-entry multivariable logistic regression predicting permanent major deficit in all surgically treated tumors.**

| **Variable** | **Univariable model** | | **Forced entry multivariable model** | |
| --- | --- | --- | --- | --- |
|  | **OR (95% CI)** | ***P* Value** | **OR (95% CI)** | ***P* Value** |
| Age (years) | 1.02 (0.99 – 1.06) | .16 | 1.03 (1.00 – 1.07) | .05 |
| Awake surgery | 1.48 (0.41 – 4.24) | .50 | 0.56 (0.14 – 1.94) | .38 |
| IONM | 3.28 (1.29 – 9.01) | **.02** | 2.98 (0.91 – 10.5) | .08 |
| Ultrasound | 1.35 (0.53 – 3.70) | .54 | 1.21 (0.46 – 3.45) | .71 |
| Advanced imaging^a^ | 3.81 (1.24 – 16.6) | **.04** | 2.87 (0.72 – 14.6) | .16 |
| Tumor volume (cm^3^) | 1.00 (0.99 – 1.01) | .42 | 1.00 (0.99 – 1.01) | .33 |
| Histopathology |  |  |  |  |
| Oligoastrocytoma | 1.41 (0.30 – 5.10) | .62 | 1.31 (0.27 – 5.03) | .71 |
| Oligodendroglioma | 1.14 (0.41 – 3.17) | .80 | 1.00 (0.34 – 2.90) | .10 |
| Astrocytoma | reference | - | reference | - |

Abbreviations: IONM, intraoperative neurophysiological monitoring and mapping

^a^Advanced imaging: functional magnetic resonance imaging (fMRI), diffusion tensor imaging (DTI), navigated transcranial magnetic stimulation (nTMS)

**D. Univariable and forced-entry multivariable logistic regression predicting permanent major deficit in all surgically treated eloquent tumors.**

| **Variable** | **Univariable model** | | **Forced entry multivariable model** | |
| --- | --- | --- | --- | --- |
|  | **OR (95% CI)** | ***P* Value** | **OR (95% CI)** | ***P* Value** |
| Age (years) | 1.00 (0.96 – 1.04) | .93 | 1.01 (0.97 – 1.06) | .53 |
| Awake surgery | 1.00 (0.27 – 3.06) | .10 | 0.51 (0.13 – 1.81) | .31 |
| IONM | 2.45 (0.81 – 9.07) | .14 | 2.23 (0.59 – 9.95) | .26 |
| Ultrasound | 1.59 (0.55 – 5.25) | .41 | 1.34 (0.44 – 4.61) | .61 |
| Advanced imaging^a^ | 5.22 (1.01 – 95.6) | .12 | 4.08 (0.61 – 82.3) | .28 |
| Tumor volume (cm^3^) | 1.00 (0.99 – 1.01) | .76 | 1.00 (0.99 – 1.01) | .93 |
| Histopathology |  |  |  |  |
| Oligoastrocytoma | 1.71 (0.34 – 6.95) | .47 | 1.92 (0.36 – 8.51) | .40 |
| Oligodendroglioma | 1.12 (0.34 – 3.69) | .85 | 1.13 (0.31 – 4.03) | .85 |
| Astrocytoma | reference | - | reference | - |

Abbreviations: IONM, intraoperative neurophysiological monitoring and mapping

^a^Advanced imaging: functional magnetic resonance imaging (fMRI), diffusion tensor imaging (DTI), navigated transcranial magnetic stimulation (nTMS)

**Supplemental Table 4.** New/worsened neurological deficits after biopsy or resection (further divided between use of IONM or not)

| **Neurological deficits, No. (%)** | **Biopsy (n=119)** | **Resection (n=398)** | **No IONM (n=256)** | **IONM (n=142)** |
| --- | --- | --- | --- | --- |
| Any new/worsened deficit^a^ | 11 (9.2) | 165 (41.5) | 65 (25.7) | 100 (70.4) |
| Any Permanent deficit^a,b^ | 3 (2.5) | 85 (21.4) | 31 (12.1) | 54 (38.0) |
| Any Permanent major deficit^a,b,c^ | 0 | 19 (4.8) | 7 (2.7) | 12 (8.5) |
| Language, new/worsened | 5 (4.2) | 76 (19.1) | 36 (14.2) | 40 (28.2) |
| Permanent | 3 (2.5) | 32 (8.0) | 14 (5.5) | 18 (12.7) |
| Permanent major | 0 | 5 (1.3) | 3 (1.2) | 2 (1.4) |
| Motor, new/worsened | 4 (3.4) | 87 (21.9) | 32 (12.6) | 55 (38.7) |
| Permanent | 0 | 36 (9.0) | 9 (3.6) | 27 (19.0) |
| Permanent major | 0 | 11 (2.8) | 4 (1.6) | 7 (4.9) |
| Facial (T/P/M) | 2(1.7)/0/0 | 17(4.3)/4(1.0)/1(0.3) | 5(2.0)/1(0.4)/0 | 12(8.5)/3(2.1)/0 |
| One limb (T/P/M)) | 1(0.8)/0/0 | 38(9.5)/10(2.5)/1(0.3) | 16(6.3)/3(1.2)/0 | 22(15.5)/6(4.2)/0 |
| Hemiparesis (T/P/M) | 1(0.8)/0/0 | 38(9.5)/24(6.0)/10(2.5) | 11(4.3)/5(2.0)/4(1.6) | 29(20.4)/19(13.4)/6(4.2) |
| SMA | 0 | 22 (5.5) | 5 (2.0) | 17 (12) |
| Permanent | NA | 8 (2.1) | 1(0.4) | 7(4.9) |
| Permanent major | NA | 0 | 0 | 0 |
| Pure Motor (T/P/M) | NA | 10(2.5)/3(0.8)/0 | 3(1.2)/0/0 | 7(4.9)/3(2.1)/0 |
| Motor + Verbal (T/P/M) | NA | 12(3.0)/5(1.3)/0 | 2(0.8)/1(0.4)/0 | 10(7.0)/4(2.8)/0 |
| Visual, new/worsened | 2 (1.7) | 19 (4.8) | 12 (4.7) | 7 (4.9) |
| Permanent | 0 | 9 (2.3) | 4 (1.1) | 5 (3.4) |
| Permanent major | 0 | 2 (0.5) | 0 | 2 (1.4) |
| Quadrantanopia (T/P/M) | 0 | 8(2)/6(1.5)/2(0.5) | 6(1.7)/3(0.8)/0 | 3(2.1)/3(2.1)/2(1.4) |
| Hemianopia (T/P/M) | 2 (1.7) | 7(1.8)/3(0.8)/0 | 4(1.1)/1(0.3)/0 | 3(2.1)/2(1.4)/0 |
| Other^d^ (T/P/M) | 0 | 3 (0.8) | 2(0.6) | 1(0.7) |
| Cognitive, new/worsened | 2 (1.7) | 20 (5.0) | 14 (5.5) | 7 (4.9) |
| Permanent | 0 | 14 (3.5) | 10 (4.0) | 4 (2.8) |
| Permanent major | 0 | 4 (1.0) | 3 (1.2) | 1 (0.7) |
|  |  |  |  |  |
| **Neurological deficits, No. (%)** | **Biopsy (n=119)** | **Resection (n=398)** | **No IONM (n=256)** | **IONM (n=142)** |
| Sensory, new or worsened | 0 | 12 (3.0) | 3 (0.8) | 9 (6.2) |
| Permanent | NA | 3 (0.8) | 1 (0.3) | 2 (1.4) |
| Permanent major | NA | 0 | 0 | 0 |
| Parietal lobe syndrome, new or worsened | 0 | 3 (0.8) | 0 | 3 (2.1) |
| Permanent | NA | 2 (0.6) | NA | 2 (1.4) |
| Permanent major | NA | 0 | NA | 0 |
| Neglect (T/P/M) | NA | 2(0.6)/1(0.3)/0 | NA | 2(1.4)/1(0.7)/0 |
| Apraxia (T/P/M) | NA | 0 | NA | 0 |
| Gerstmann syndrome (T/P/M) | NA | 0 | NA | 0 |
| Spatial orientation (T/P/M) | NA | 1(0.3)/1(0.3)/0 | NA | 1(0.7)/1(0.7)/0 |

Abbreviations: IONM, intraoperative neurophysiological monitoring and mapping; SMA, supplementary motor area; NA, Not applicable; T, total new or worsened deficit; P, permanent deficit; M, permanent major deficit.

^a^Patient wise. All neurological deficits are reported, resulting in a higher total number than reported under the compilation any new/worsened or permanent deficit, since the same patient can experience more than one type of deficit.

^b^Permanent deficit, deficit persisting beyond 3 months after biopsy or resection.

^c^Permanent major deficit, i.e., having impact on daily life.

^d^Other visual deficit: gaze palsy=1, oculomotor nerve palsy=1, scotoma=1.

**Supplemental Table 5.** Permanent neurological deficits (persisting >3 months) after resection, by neurological function monitored.

|  | **Functions monitored with IONM^a^** | **Functions not monitored** | ***P* Value** |
| --- | --- | --- | --- |
| Any neurological function monitored, No. | 142 | 256 |  |
| Any permanent neurological deficits, No. (%) | 54 (38.0) | 31 (12.1) | <.001 |
| Any permanent major deficits^b^, No. (%) | 12 (8.5) | 7 (2.7) | .01 |
| New seizures^c^, No. (%) | 13 (9.2) | 8 (3.2) | .02 |
| Language, No. | 43 | 355 |  |
| Permanent language deficits, No. (%) | 8 (17.8) | 23 (6.5) | .02 |
| Permanent major deficits, No. (%) | 0 | 5 (1.4) | 1.0 |
| Motor, No. | 115 | 283 |  |
| Permanent motor deficits, No. (%) | 27 (27.8) | 9 (3.2) | <.001 |
| Permanent major deficits, No. (%) | 7 (6.1)^d,e^ | 4 (1.4) | .02 |
| Sensory, No. | 20 | 378 |  |
| Permanent sensory deficits, No. (%) | 1 (5.0) | 2 (0.5) | .15 |
| Permanent major deficits, No. (%) | 0 | 0 |  |
| Other parietal, No. | 2 | 396 |  |
| Permanent other parietal deficits, No. (%) | 2 (100) | 2 (0.5) | 1.0 |
| Permanent major deficits, No. (%) | 0 | 0 |  |
| Visual, No. | 6 | 392 |  |
| Permanent visual deficits, No. (%) | 1 (16.7) | 8 (2.0) | .13 |
| Permanent major deficits, No. (%) | 0 | 2 (0.5) | 1.0 |

Abbreviations: IONM, intraoperative neurophysiological monitoring and mapping

^a^Several indications possible in each patient.

^b^Permanent major deficit, i.e. having impact on daily life

^c^New onset seizures or status epilepticus after surgery.

^d^Awake surgery and exclusively asleep mapping for 4 and 3 patients, respectively, with no statistically significant difference of frequency of deficits between monitoring modality.

^e^Stopped resection due to: functional limits in 5 patients, perceived Gross Total Resection in 1 patient.

**Supplemental Table 6.** Adverse events within 30 days, in patients undergoing resection (divided by use of IONM or not), according to Landriel Ibanez classification.

|  | **Resection (n=398)** | **No IONM (n=256)** | **IONM (n=142)** | ***P* Value** |
| --- | --- | --- | --- | --- |
| Any complication, No. (%)^a^ | 87 (21.9) | 51 (20.2) | 36 (25.4) | .26 |
| Grade I |  |  |  | .12 |
| New onset seizure / status ep | 21 (5.3) | 8 (3.2) | 13 (9.2) | .02 |
| Worsened seizures | 5 (1.3) | 4 (1.6) | 1 (0.7) |  |
| Urinary tract infection | 3 (0.8) | 2 (0.8) | 1 (0.7) |  |
| Pneumonia | 6 (1.5) | 4 (1.6) | 2 (1.4) |  |
| Superficial wound infection | 9 (2.3) | 3 (1.2) | 6 (4.2) |  |
| Cerebral infarction | 4 (1.0) | 4 (1.6) | 0 |  |
| Grade II |  |  |  | .25 |
| Deep wound infection | 13 (3.3) | 7 (2.8) | 6 (4.2) |  |
| Meningitis | 2 (0.5) | 2 (0.8) | 0 |  |
| CSF leakage | 2 (0.5) | 2 (0.8) | 0 |  |
| Deep vein thrombosis | 3 (0.8) | 3 (1.2) | 0 |  |
| Pulmonary embolism | 4 (1.0) | 2 (0.8) | 2 (1.4) |  |
| Endocrinological disturbance | 4 (1.0) | 2 (0.8) | 2 (1.4) |  |
| Cardiac disturbance | 1 (0.3) | 0 | 1 (0.7) |  |
| Grade III |  |  |  | .75 |
| Epidural hematoma | 11 (2.8) | 5 (2.0) | 6 (4.2) |  |
| Resection cavity hematoma | 7 (1.8) | 5 (2.0) | 2 (1.4) |  |
| Other hematoma | 4 (1.0) | 4 (1.6) | 0 |  |
| Hydrocephalus | 2 (0.5) | 2 (0.8) | 0 |  |
| Cerebral infarction | 1 (0.3) | 1 (0.4)) | 0 |  |
| Grade IV^b^ | 1 (0.3) | 1 (0.4) | 0 | 1.0 |
| Other^c^ | 7 (1.8) | 3 (1.2) | 4 (2.8) | .43 |
|  |  |  |  |  |
|  |  |  |  |  |
|  |  |  |  |  |
|  | **Resection (n=398)** | **No IONM (n=256)** | **IONM (n=142)** | ***P* Value** |
| Most severe complication, No. (%) | | | |  |
| Grade I | 51 (12.8) | 28 (11.1) | 23 (16.2) | .30 |
| Grade II | 21 (5.3) | 11 (4.3) | 10 (7.0) |  |
| Grade III | 11 (2.8) | 8 (3.2) | 3 (2.1) |  |
| Grade IV | 1 (0.3) | 1 (0.4) | 0 |  |

Abbreviations: CSF, cerebrospinal fluid; IONM, intraoperative neurophysiological monitoring and mapping; status ep, status epilepticus

^a^Patient wise. All complications are reported under the different gradings, resulting in a higher total number than reported under any complication and most severe complication, since the same patient can experience more than one complication.

^b^Death due to urosepsis.

^c^Other complications (Urinary retention, transient loss of consciousness, miscarriage, other infection, fever, edema, bone flap removal)

**Supplementary Fig. 1.** Boxplot of Extent of Resection (EOR) by patient center.


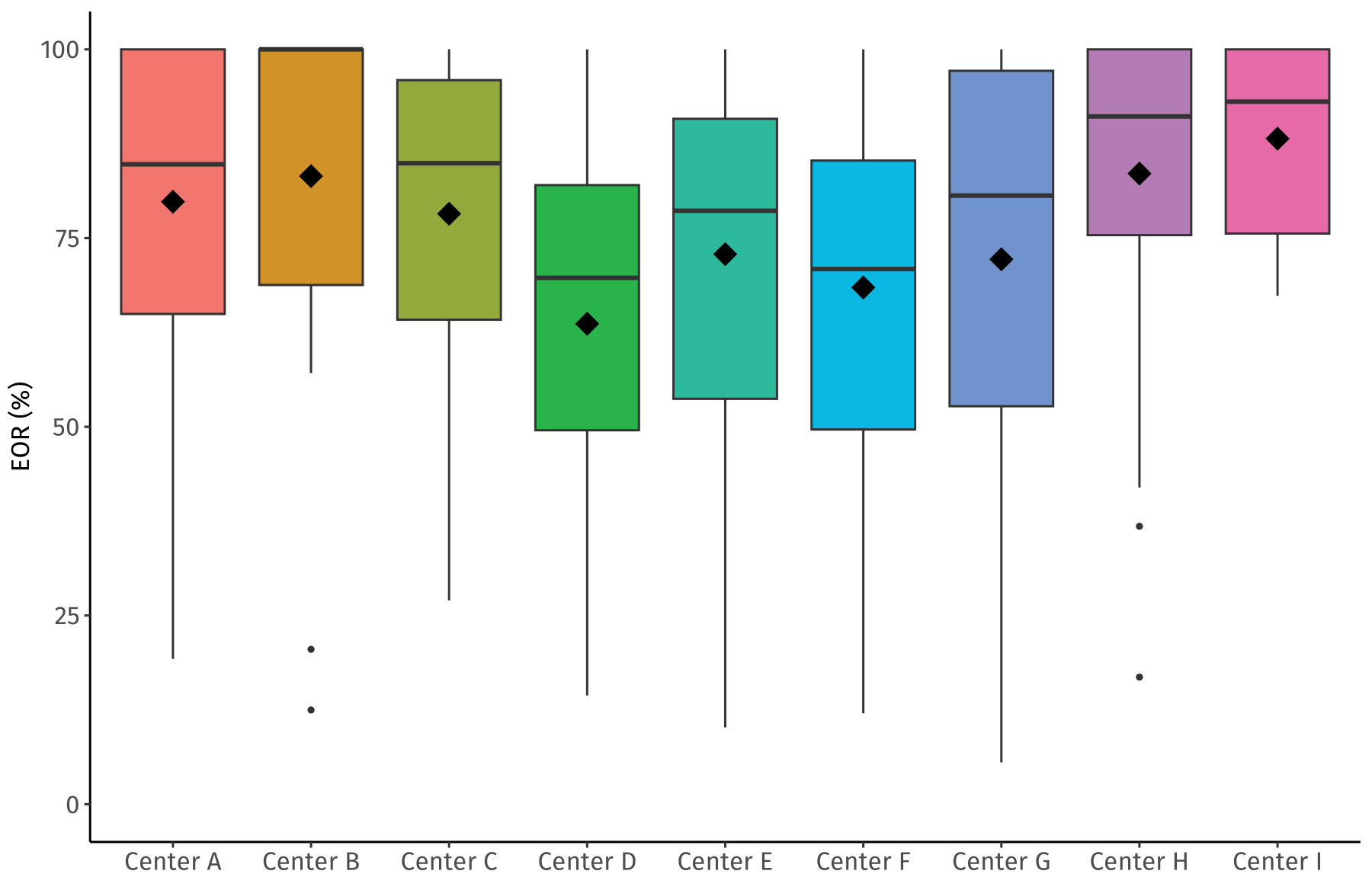


The patient centers are represented with a letter and different colors. Within each box the horizontal line represents median EOR and the black diamond the mean: Boxes extend form the 25^th^ to the 75^th^ percentile of each groups values (IQR, interquartile range). Vertical lines denote values within 1.5 IQR and dots denote observations without this range.

**Supplementary Fig. 2.**


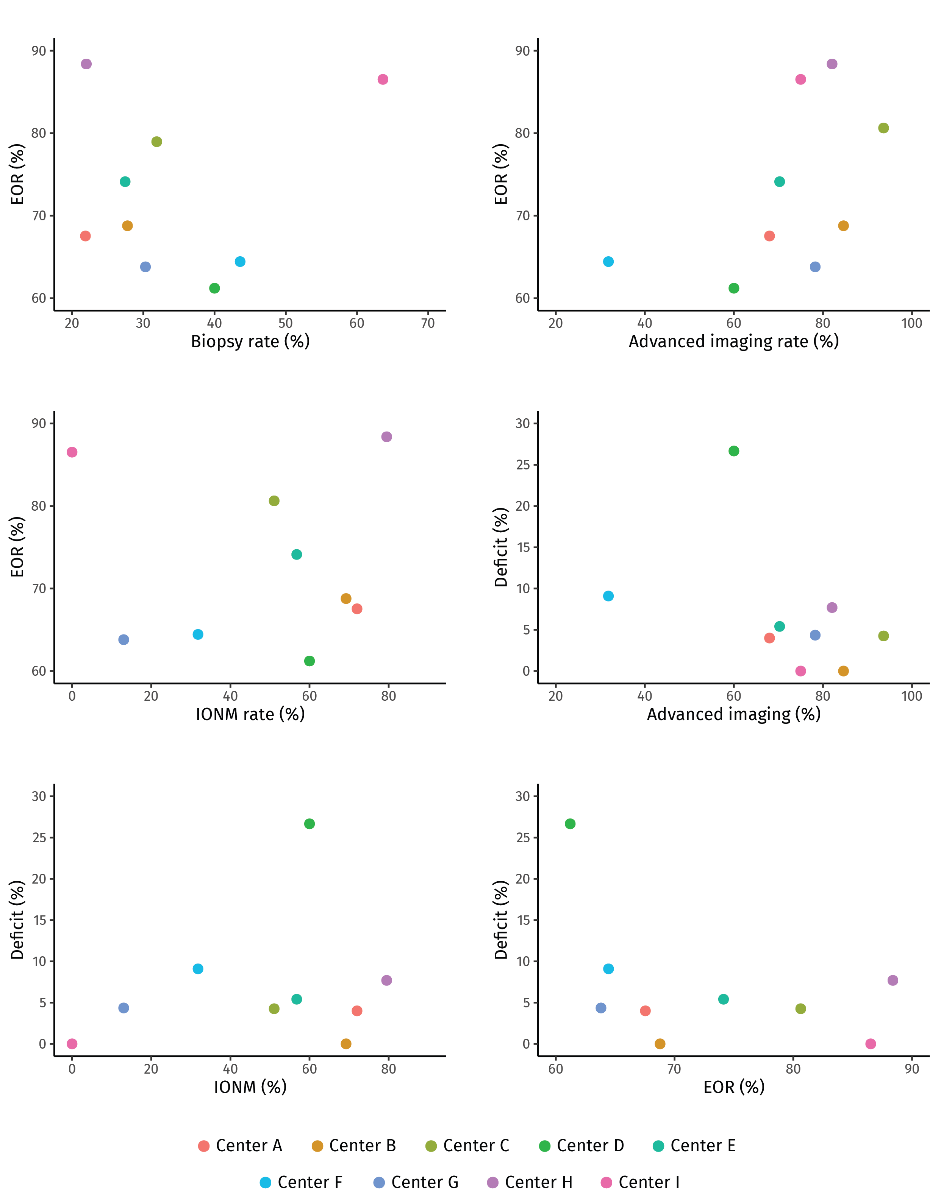


Scatterplots displaying relationship between key outcome parameters in resected tumors in presumed eloquent area, by patient centers (dots). Each center is represented with a different color. (A) The relationship between proportion of biopsy and median EOR. (B)The relationship between proportion of advanced imaging and median EOR. (C) The relationship between proportion of IONM and median EOR. (D) The relationship between proportion of advanced imaging and proportion of permanent major neurological deficit. (E) The relationship between proportion of IONM and proportion of permanent major neurological deficit. (F) The relationship between median EOR and proportion of permanent major neurological deficits.

**Supplementary Fig. 3**. Oncological and functional outcome based on case volume and use of IONM.


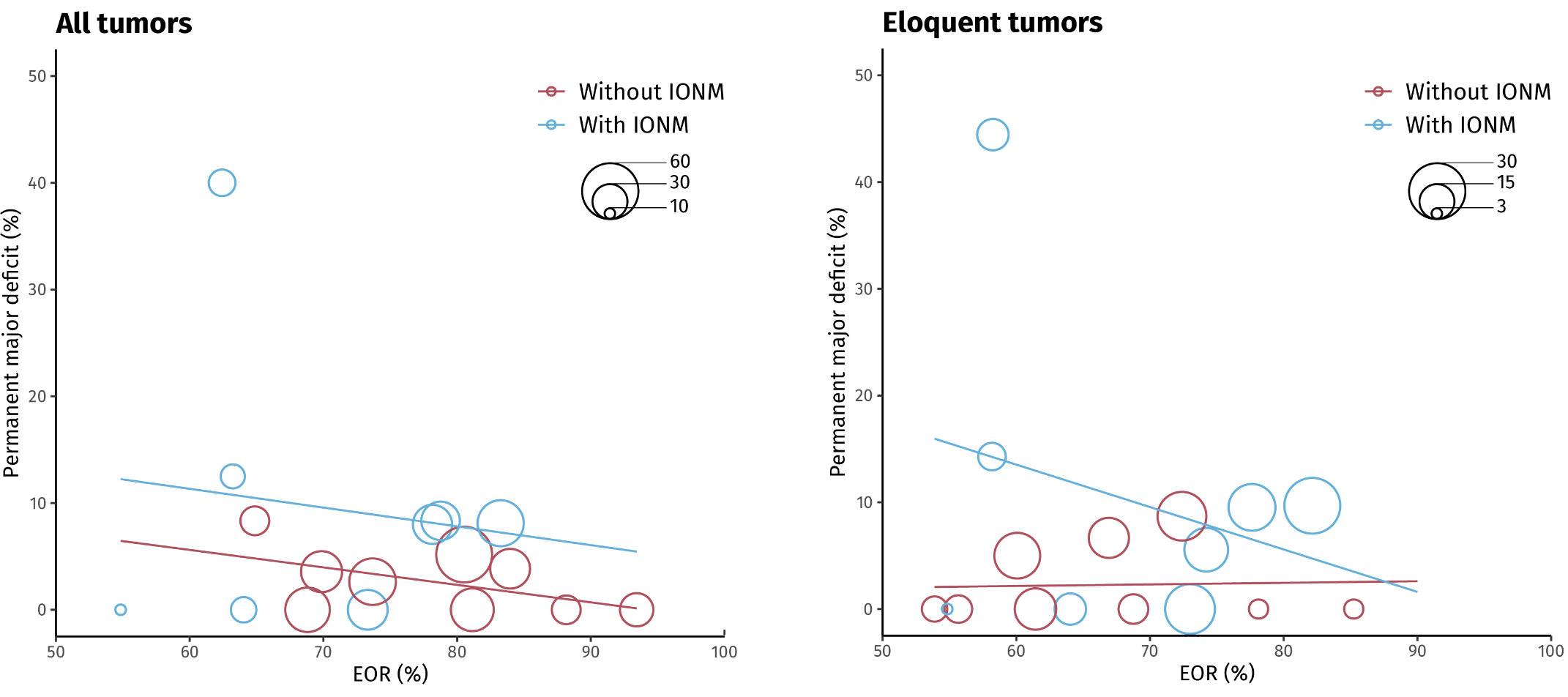


Bubble charts displaying the relationship between EOR (Extent of resection) and postoperative neurological deficits by patient center and use of IONM (Intraoperative neurophysiological monitoring and mapping) The percentage of EOR versus percentage of permanent major deficits plotted, for all tumors and tumors in a presumed eloquent area and grouped by resection with or without IONM. Each dot represents a patient center, and the dot size represents case volume at the respective center (the scale for dot size is shown in figure legend).
